# Characterization of the complete chloroplast genome of mangrove *Bruguiera gymnorrhiza (*L.) Lam. ex Savigny

**DOI:** 10.1080/23802359.2021.1914220

**Published:** 2021-06-23

**Authors:** Su-Yuan Li, Yi-Chan Li, Tian-Hao Zhang, Lan-Li Qin, Yi-Dong An, Yu-Kun Pang, Guo-Feng Jiang

**Affiliations:** Guangxi Key Laboratory of Forest Ecology and Conservation and State Key Laboratory for Conservation and Utilization of Subtropical Agro-Bioresources, College of Forestry, Guangxi University, Nanning, PR China

**Keywords:** Mangrove, Bruguiera, plastid genome, evolution

## Abstract

The chloroplast (cp) genome sequence of *Bruguiera gymnorrhiza* was characterized. The cp genome length was 163,795 bp in length, with a GC content of 35.3%, containing a large single copy (LSC) of 90,830 bp, a small single copy (SSC) of 20,207 bp, and a pair of inverted repeats (IRs) of 26,379 bp. The genome contained 121 genes, including 84 protein-coding genes, 37 tRNA genes, and 8 rRNA genes. A phylogenetic analysis using cp genomes of mangroves and ecologically associated species resolved *B*. *gymnorrhiza* in *Bruguiera* with *B. sexangula* var*. rhynchopetala.* This complete chloroplast sequence offers a promising tool for further species identification and evolutionary studies of *Bruguiera*, as well as for mangroves.

## Introduction

Mangroves are a diverse group of about 70 woody trees and shrubs that inhabit the coasts of tropical and subtropical regions (Ball 1988; Duke 1992). Despite the seemingly harsh environment, mangrove ecosystems are highly productive ecosystems with rates of primary production equal to those of tropical humid evergreen forests and coral reefs (Barr et al. 2010; Alongi 2014; Lu et al. 2017). Nowadays mangroves are threatened by climate changed-induced drought, as well as, relative sea level rise (Lovelock 2020; Saintilan et al. 2020). Mangrove forests have been severely degraded over the past half century (Blasco et al. 2001; Donato et al. 2011), injecting new urgency into understanding the genetic resources of mangroves.

Large-leafed mangrove *Bruguiera gymnorrhiza* is one of the most important and widespread mangrove species, and widely distributed from the eastern coast of Africa through Asia to subtropical Australia (Allen and Duke 2006). The wood of *B*. *gymnorrhiza* is widely used for structural components of traditional homes, while people also using *B*. *gymnorrhiza* for food, as well as for dyes and traditional medicines (Allen and Duke 2006). Therefore, providing the genome sequences of species *B*. *gymnorrhiza* will help to spur research on these most interesting adaptations, and also could offer some needed information for its usage and conservation. Chloroplast DNA (cpDNA) have been proved could provide useful and abundant information on genetic diversity and evolution based on our previous studies (Jiang et al. 2016; Lei et al. 2018), as well as in mangroves (Chen et al. 2019; Yang et al. 2019; Shi et al. 2020). In this study, we assembled and characterized the chloroplast genome of *B*. *gymnorrhiza* based on Illumina pair-end data, and built a phylogenetic tree using plastomes available in mangroves and ecologically associated species.

*B*. *gymnorrhiza* was collected in Sanya Tielu Port mangrove reserve (Sanya, PR China, 18° 15′ N/109° 42′ E). The voucher (*B*. *gymnorrhiza*_Jiang_B7) is stored in Guangxi University, plant ecophysiology and evolution research group herbarium. Total genomic DNA was extracted from 0.1 g of frozen fresh leaves as previously described (Jiang et al. 2016; Lei et al. 2018; Xu et al. 2018). A 350-bp paired-end library was constructed and sequenced by Novogene (Beijing, PR China) using an Illumina HiSeqX-ten system (Illumina, San Diego, CA), about 1 Gb raw data filtered for read quality was obtained. We performed a *de novo* assembly using NOVOPlasty3.6 (Dierckxsens et al. 2016), seed sequence from mangrove *Avicennia marina* was retrieved from NCBI under the accession number (AB114520.1). The assembled sequence was then imported into Geneious R9 (Biomatters Ltd, Auckland, New Zealand), to check manually as described previously (Jiang et al. 2016; Hinsinger and Strijk 2017; Xu et al. 2017). The cp genome annotation was transferred from *Rhizophora* x *lamarkii* (NC_046517), a species from Rhizophoraceae. The final annotations were confirmed and integrated from results of CPGAVAS2 (Shi et al. 2019) and Chloroplot (Zheng et al. 2020).

The assembled cp genome of *B*. *gymnorrhiza* had a length of 163,795 bp (GenBank accession number MW402841). The cp genome exhibited the typical composition of LSC, SSC regions and two inverted repeats (IRa and IRb) of 90,830, 20,207, and 26,379 bp, respectively. The overall GC content of the plastome of *B*. *gymnorrhiza* was 35.3%, while the GC content in LSC, SSC, IRa, and IRb regions were 32.9%, 28.0%, 42.2%, respectively. We identified 121 genes, including 84 protein-coding genes, 37 tRNA genes, and 8 ribosomal RNA genes. Thirteen genes (*atpF*, *petB*, *petD*, *rpoC1*, *trnK*-UUU, *trnL*-UAA, *trnT*-CGU, *trnV*-UAC in LSC; *ndhA* locates in SSC; *ndhB*, *rpl2*, *trnA*-UGC, *trnI*-GAU in the IRs regions) contain 1 introns; while *clpP*, *ycf3* in LSC, and *rpS12* in IRb contain 2 introns, respectively. In total, 18 genes were duplicated in the IR regions, including 8 protein-coding genes (*rps19*, *rpl2*, *rpl23*, *ycf1*, *ycf2*, *ndhB*, *rps7*, *rps12*), 7 tRNA genes (*trnA-UGC*, *trnI-GAU*, *trnI-CAU*, *trnL-CAA*, *trnN-GUU*, *trnR-ACG*, *trnV-GAC*), and 4 rRNA genes (4.5S, 5S, 16S, 23S). Interestingly, *B*. *gymnorrhiza* had its own codon usage bias in comparison to *Barringtonia racemosa* from the analysis by Chloroplot (Zheng et al. 2020), especially large differences were found in *psbK*, *ndhG*, and *rps7*.

**Figure 1.**
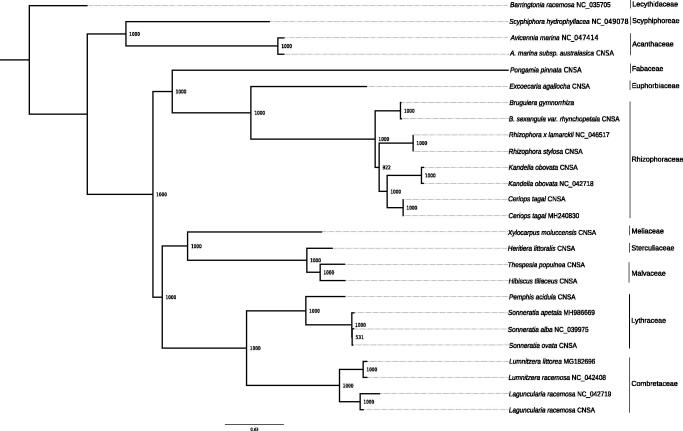
ML phylogeny of mangroves and ecologically associated species based on 25 cp genomes retrieved from the present study, GenBank and CNSA. The tree is rooted with *Barringtonia racemosa*. Bootstraps values (1000 replicates) are shown at the nodes. Scale in substitution per site.

Twenty-four plastomes of mangroves and ecologically associated species were retrieved from GenBank (accessed 17 November 2020), and CNSA (https://db.cngb.org/cnsa/) according to the reference (Shi et al. 2020), plus *Barringtonia racemosa* as an out-group (Figure 1). All the cps were aligned with MAFFT v7.307 (Katoh and Standley 2013), and a maximum likelihood (ML) tree was built using PhyML v3.3 (Guindon et al. 2009) with a GTR + I + G model and support estimated with 1000 bootstrap replicates. All but one node were highly supported (bootstrap support ≥92), with the two species of *Bruguiera* clustered together. The plastome of *B*. *gymnorrhiza* provides a useful bioresource that will help to assess population diversity for conservation purposes, and will also benefit to further genetic studies in Rhizophoraceae.

## Data Availability

The genome sequence data that support the findings of this study are openly available in GenBank of NCBI at (https://www.ncbi.nlm.nih.gov/) under the accession no. MW402841. The associated BioProject, SRA, and Bio-Sample numbers are PRJNA713533, SRR13933381, and SAMN18253765, respectively. Data were also available in the database: CNGB Sequence Archive (CNSA) of China National GeneBank DataBase (CNGBdb) with accession number CNP0001525 (https://db.cngb.org/search/project/CNP0001525/).
